# Case report: Mononeuritis multiplex in the course of dengue fever

**DOI:** 10.1186/s12879-020-05430-8

**Published:** 2020-09-22

**Authors:** Jun Yang Ho, Yee Kent Liew, Jiashen Loh, Pothiawala Sohil

**Affiliations:** Seng Kang General Hospital, Singapore, Singapore

**Keywords:** Diplopia, Mononeuritis multiplex, Dengue, Abducens nerve, Foot drop

## Abstract

**Background:**

Dengue fever usually presents as a self-limiting acute febrile illness with worsening thrombocytopenia, with a small minority of patients developing hemorrhagic or life-threatening complications. Organ specific manifestations like myocarditis, acalculous cholecystitis, encephalitis has been described but are uncommon presentations. Even more rarely, such manifestations are the presenting complaint of Dengue fever. In this case report, we highlight a case of Dengue fever where unrelated neuropathies were the presenting complaint.

**Case presentation:**

An elderly man presents with 1 day of diplopia and left foot drop, associated with 2 days history of fever. A decreasing white cell count (WBC) and platelet on the 2nd day of admission prompted Dengue virus to be tested and a positive NS-1 antigen was detected, confirming the diagnosis of Dengue fever. He was treated with supportive treatment with a short duration of intravenous fluids recovered uneventfully and was discharged 6 days after admission with almost full resolution of diplopia and partial resolution of left foot drop. Left foot drop recovered completely 2 weeks later.

**Conclusion:**

Neurological manifestations can be the presenting symptoms in Dengue fever, a diagnosis which should be borne in mind when such symptoms present in patients from endemic areas or in returning travellers from these areas.

## Background

Dengue fever is a vector-borne infection caused by the dengue viruses of the Flaviviridae family, which consists of four serotypes (DenV-1, DenV-2, DenV-3 and DenV-4). It is estimated that there are around 390 million dengue infections per year [1] spanning across 128 countries [2]. The major burden of this disease, nearly 75%, is borne by the South-East Asian region and the Western Pacific region. The estimated fatality rate is about 1% in the South-East Asia region [3].

While dengue fever commonly presents with febrile illness and no localizing source of infection, it can manifest with neurological symptoms. Dengue guidelines released by WHO in 2019 incorporated central nervous involvement in the definition of severe disease [3]. However, no neurological features have been specifically associated with dengue fever. There have been reported cases of patients who present with neurological symptoms and dengue virus was isolated from their cerebrospinal fluid, suggesting that the virus crosses the blood-brain barrier and directly invades the brain causing encephalitis [4] [5]. With increasing evidence of dengue neurotropism, an effort was made to categorize dengue related neurological features into 4 groups, namely, dengue encephalopathy, encephalitis, neuromuscular complication and neuro-ophthalmic involvement [6].

While dengue encephalitis and encephalopathy are commonly reported, very few cases of mononeuropathies have been reported in the past. There have been cases that involved isolated facial nerve palsy [7–9], long thoracic nerve palsy [10], phrenic nerve palsy [11, 12] and oculomotor nerve palsies [13, 14]. The pathogenesis behind dengue related mononeuropathies is thought to be immune-mediated.

We report a case of right abducens nerve palsy and left foot drop secondary to dengue fever.

## Case presentation

A 71-year male presented to the Emergency department (ED) complained of diplopia on waking up in the morning and weakness over his left lower limb on the day of his ED visit. He also reports 2 days of fever. There is no left lower limb pain. He has no headache. He denied any travel history. However, he often walked around his neighbourhood to play Pokemon Go (an augmented reality mobile phone game which requires gamers to roam widely to capture virtual avatars). He reports allergy to aspirin, does not drink alcohol and is a smoker of 60 pack years. Systems review was unremarkable. He had a past medical history of diabetes mellitus with a HbA1c of 8% 2 months before presentation, hypertension, dyslipidaemia, ischemic heart disease with good effort tolerance after a percutaneous coronary intervention 8 years ago, stage four chronic kidney disease and abdominal aortic aneurysm with endovascular aneurysm repair 7 years ago. He was on furosemide, valsartan, glipizide, bisoprolol, nifedipine, simvastatin, metformin, clopidogrel on admission.

**Fig. 1:**
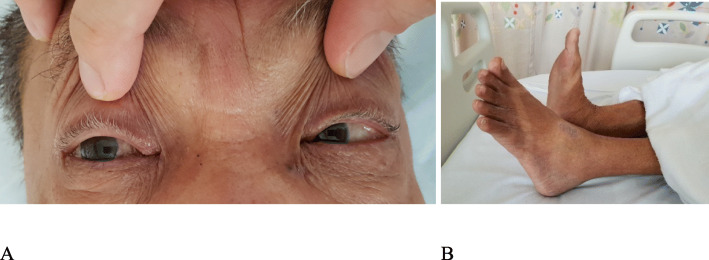
**a** Right abducens nerve palsy and (**b**) left foot drop on the day of admission

At ED, he was febrile at 38.1 °C, blood pressure was 121/59 mmHg and heart rate was 68 beats per minute. Physical examination revealed dual heart sounds, clear lung fields on auscultation, soft and non-tender abdomen on palpation, normal bowel sound. Neurological examination revealed right lateral rectus palsy, suggestive of an isolated right sided VIth cranial nerve palsy, and a left foot drop (Fig. 1). Horizontal diplopia was maximal at right gaze and diplopia resolves on closing either eye. Bilateral left ankle jerk reflex were absent. Bilateral knee jerks were present. Upper limb tendon reflexes were present. Motor power of all four limbs was equal, except for left ankle dorsiflexion. Sensation of all dermatomes was normal. There was no rash or palpable lymphadenopathy.

Blood investigations done in the ED were normal except for an elevated C-reactive protein of 48.3 mg/L and procalcitonin of 1.82μg/L. His renal function showed creatinine of 380 umol/L (baseline creatinine 260umol/L) and urea of 17.9 umol/L. There was no consolidation/effusion on the chest radiograph but a right lower lobe lung nodule was noted. Computed tomography of the brain done in the ED showed no acute lesion to account for his symptoms, except for chronic microvascular ischaemic changes. He was given intravenous antibiotics and then admitted for further management.

In the ward, no new symptoms or signs were elicited. Given this patient’s smoking history and opacity on chest X-ray, an initial diagnosis of paraneoplastic mononeuritis multiplex was considered. IV augmentin was started empirically in response to fever and high procalcitonin. Repeat full blood count 1 day after admission showed thrombocytopenia (134 × 10^9^/l) with leukopenia (3.80 × 10^9^/l) suggestive of dengue fever. Dengue serology and antigen test was negative for Dengue IgM and IgG but positive for NS1 antigen. MRI brain showed chronic lacunar infarcts. Anti-MPO and anti-PR3 antibodies, cryptococcal antigen and HIV screen were negative. Tumor marker screening (carcinoembryonic antigen, Ca125, Ca15–3, Ca 19–9, alpha-fetoprotein, prostate specific antigen) showed slightly elevated CEA of 8.6μg/L and PSA of 4.46μg/L Rest of the markers were negative. Four hourly vital signs monitoring were started with twice daily postural blood pressure monitoring. IV fluids were started on admission and stopped on day 2, when he gained sufficient appetite for oral hydration and nutrition. Clopidogrel was stopped on day 2 of admission when Dengue NS1 was positive, in anticipation of impending thrombocytopenia. When blood cultures did not yield any bacterial growth 2 days after admission, IV augmentin was stopped. Defervescence occurred 3 days after admission. There was no postural hypotension during his admission. No corticosteroids were administered. Daily full blood count documents a worsening thrombocytopnenia, reaching a platelet nadir of 24 × 10^9^/l at day 6 of admission and subsequently a recovering WBC and platelet trend on day 7. Detailed values of important blood investigations are found in the attached supplementary material. He recovered fully from dengue fever without further complications. There was no shock or hemorrhagic manifestation throughout his disease course. He was discharged on day 7 post admission after some physical therapy. Two weeks after discharge, his platelet was normalized at 503 × 10^9^/l. His final diagnosis was mononeuritis multiplex secondary to dengue fever.

**Fig. 2:**
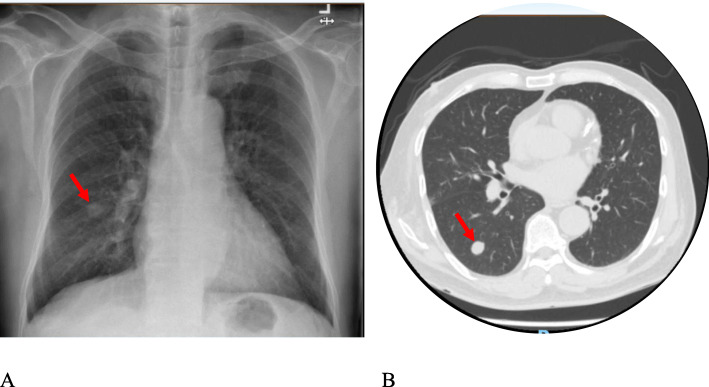
**a** chest x-ray and (**b**) CT Thorax

**Fig. 3:**
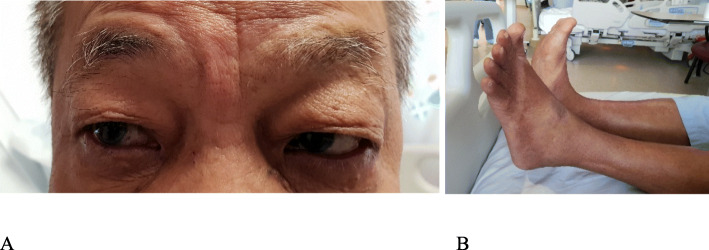
**a** Resolved right abducens nerve palsy and (**b**) persistent left foot drop upon discharge

CT thorax was done to evaluate the lung nodule seen on chest X-ray. CT thorax showed a nodule in the right lower lobe, suspicious for a primary neoplasm (Fig. 2). His right CN VI palsy completely resolved after 5 days but the foot drop persisted, though significantly improved. (Fig. 3). Due to thrombocytopenia, a lung biopsy was scheduled for a later date. He was then discharged from the hospital after 7 days of admission. Outpatient follow-up notes showed that his left foot drop completely resolved after 2 weeks, further follow-up up to 8 months after presentation showed no recurrence. The lung nodule was subsequently proven to be adenocarcinoma on histology.

## Discussion and conclusion

This case highlights an uncommon presentation of Dengue fever manifesting early as the presenting symptom. We think that such uncommon presentation deserves attention as they may result in diagnostic delays. We were unable to send the test for PCR as it is not part of routine clinical care. Hence, we were unable to determine and report the which serotype of Dengue virus is responsible. All serotypes circulate in Singapore, the predominant strains being DenV 1 and DenV 2.

Several pathogenic mechanisms have been hypothesized to contribute to the neurological manifestations of the disease. Up till 2012, 3 pathogenic mechanisms of the neurological complications of dengue infection were described [15]; 1) direct invasion of the CNS by the virus causing neurotropic effects like meningitis, encephalitis, myositis. 2) metabolic abnormalities resulting in CNS complications such as encephalopathy, stroke and hypokalaemic paralysis and 3) autoimmune complications including encephalomyelitis, Guillain-Barré syndrome (GBS), cranial nerve palsy [16] and optic neuritis [17]. All 4 serotypes of dengue (DenV-1 to DenV-4) have been implicated in neurological manifestations of dengue fever [18–20].

The dengue virus was not classically thought to be a neurotropic entity [21]. However, recent evidence demonstrating neurotropism and CNS invasion in Dengue infection has emerged. Dengue viral antigens have been isolated in human brain tissue via immunohistochemistry in cases of fatal dengue infection [20]. DenV RNA has also been found in the CSF and cerebral tissue of patients [22], implying that the cause of dengue encephalitis is due to the direct invasion of the CNS by the virus rather than a passive crossing of the virus into the CNS secondary to a vascular leak or rupture of the blood-brain barrier.

Immune-mediated reactions such as Guillian-Barre syndrome (GBS), mononeuropathies, acute disseminated encephalomyelitis (ADEM) and brachial neuritis can also occur after DENV infection due to the formation and deposition of immune complexes in the CNS [4]. Areflexic flaccid ascending tetraparesis has been observed in a patient following Dengue infection, suggesting acute polyradiculoneuritis [23]. In that case report, same patient was also found to have a primarily demyelinating polyradiculoneuropathy with associated axonal components on electroneuromyography [23].

Patients with ADEM post Dengue infection have also been found to have demyelination with haemorrhagic foci [24] on MRI of their brains. Other MRI findings included white matter lesions in other regions of the brain such as corona radiata, callosal-septal interface and the thalamus [25]. The abovementioned constellation of findings would suggest an immune-mediated pathogenetic mechanism towards myelin or other CNS antigens, possibly via non-specific auto-reactive T cell clone activation [15], or by molecular mimicry [26].

In this report, we present an uncommon neurological complication of dengue disease, mononeuritis multiplex, manifesting as an isolated cranial nerve palsy (right abducens nerve) and a unilateral (left sided) foot drop. This complication occurred on day 2 of infection.

To our knowledge, foot drop as a complication of Dengue infection has only been described in a single publication by medical department of the US Navy [27], analysing the complications of dengue fever following an outbreak in the Central Pacific in 1924. Over a 40-day period, a total of 1488 dengue cases were treated, of which, 13 were documented as having mononeuropathies as a neurological manifestation of dengue fever. Of these 13 patients, 4 of them had peroneal nerve palsies. The other nerves affected included the facial, palatal, long thoracic and ulnar nerves [27]. In no case was there a history of recent illness or trauma to suggest another cause for any of these neurological findings. Unfortunately, there was also no documentation as to whether nerve conduction studies were performed on these patients.

Of note, these presentations of peroneal nerve palsy manifested between 2 to 5 weeks after infection. These patients were treated with thiamine chloride. However, there was no record of symptomatic improvement. These patients were subsequently evacuated from the hospital and no further information of their recovery was available [27].

In our patient, peroneal nerve palsy occurred on the 2nd day of his fever. We did not perform nerve conduction study (NCS) prior to discharge because we expect more significant positive findings if it would be performed some time after onset of symptoms and also because of severe thrombocytopenia and recent clopidogrel use. He was given an outpatient appointment for NCS, which he defaulted.

Cranial nerve palsies secondary to Dengue are also uncommon, with just a few cases reported in medical literature [28, 29]. Cranial nerves affected include the optic nerve, oculomotor nerve, facial nerve and abducens nerve [28].

Our patient and the one reported by Mazliha et al*.* [30] both developed abducens nerve palsy on day 2 of the onset of fever. Given the uncommon presentation of the patient, various other causative mechanisms were considered in addition to Dengue infection, such as diabetes related mononeuritis multiplex and paraneoplastic mononeuritis multiplex. However, his spontaneous recovery renders paraneoplastic mononeuritis multiplex unlikely. We were unable to conclusively disprove diabetes mellitus as an etiology but the immediate temporal relation with a positive dengue diagnosis makes the case compelling. We did not perform a lumbar puncture as it was not deemed beneficial to patient care, given the added risk his thrombocytopenia presents.

Given that his symptoms manifested at an early stage of his illness (day 2) and full recovery without immune modulation, direct viral invasion of cranial nerves and peripheral nerves would be the more likely pathological mechanism to account for his presentation instead of an immune-mediated mechanism.

In conclusion, Dengue remains as the world’s most common mosquito-borne viral disease to date, with an estimated incidence rate of 50 to 100 million people yearly and a mortality rate of 25,000 yearly [25]. While neurological manifestations are uncommon, they should always be considered as a possible indication of DENV infection in patients presenting with fever and acute neurological abnormalities in dengue endemic regions of the world. A wide spectrum of neurological manifestations are possible and early clinical suspicion remains key to the diagnosis in endemic areas.

## Data Availability

Data and materials for this work were obtained retrospectively from electronic clinical chart of the patient. Important clinical information is provided in the manuscript and the attached file labelled: additional clinical data.

## Abbreviations

CNS: Central nervous system
CT: Computed tomography
CN: Cranial nerve
IV: Intravenous
MRI: Magnetic resonance imaging
NCS: Nerve conduction study
